# ITC-MNP: a diverse dataset for image file fragment classification

**DOI:** 10.1186/s13104-024-07034-w

**Published:** 2024-12-19

**Authors:** Behnam Tavassoli, Zhino Naghshbandi, Mehdi Teimouri

**Affiliations:** Information Theory and Coding (ITC) Laboratory, University of Tehran, Tehran, Iran

**Keywords:** File fragment classification, File type identification, Image file fragment, Dataset

## Abstract

**Objectives:**

Image file fragment classification is a critical area of study in digital forensics. However, many publicly available datasets in this field are derived from a single source, often lacking consideration of the diversity in image settings and content. To demonstrate the effectiveness of a given methodology, it is essential to evaluate it using datasets that are sampled from varied data sources. Therefore, providing a sufficiently diverse dataset is crucial to enable a realistic assessment of any proposed method.

**Data description:**

The dataset includes image file fragments of 4096 bytes from five formats (JPG, BMP, GIF, PNG, and TIFF), each processed with different conversion settings. The source images are categorized into three content types: Nature, People, and Medical. In total, the dataset contains 501,000 fragments. These fragments consist of file headers and incomplete end-of-file fragments, completed with random bytes to approximate how operating systems handle data when file sizes are not multiples of the sector size. This approach aims to simulate typical scenarios where fragments are recovered from a hard drive, though it may not capture all real-world complexities such as data corruption and complex file structures.

## Objective

Hard drives store data in fragments, with the size of each fragment determined by the file system. Fragments from a single image can be scattered across the hard drive. In digital forensics, for instance, recovering image data from a damaged hard drive, even partially, is often necessary. This challenge is one of the reasons researchers study image file fragment classification [1]. Recent studies in this field frequently use machine learning to classify file fragments [2–4]. A diverse dataset is crucial for effectively training and evaluating these models. Our dataset aims to include a wide range of image file fragments, varying in both the content and conversion settings of the source images.

Most available datasets in this area tend not to emphasize the source images from _which_ fragments are collected. This raises several questions: Is it beneficial to consider the content of the source files? Could it influence performance if the dataset includes source files with varying settings, such as compression? And how significant might the impact of these factors be on the performance of classifiers?

Our dataset includes fragments from the five most common image file formats, which are also present in the FFT-75 [5] and Fakouri and Teimouri’s [6] datasets. This dataset is expected to help partially address the aforementioned questions and offer useful insights to experts in the field. It also facilitates the evaluation and comparison of model performance by providing a more diverse and carefully curated dataset. It will also support the evaluation and comparison of model performance by offering a more diverse and carefully curated dataset. The results section of our supporting document presents findings that illustrate the potential effectiveness of this dataset.

## Data description

In the first step, we downloaded source images from the internet and categorized them into three content groups: Nature, People, and Health. A total of 500 images were randomly selected from each category. We aimed to obtain high-quality images within each group. Detailed information about the source images is provided in the supporting document. The subsequent steps of our work are as follows:

### Choosing settings

Each of the five formats (JPEG, GIF, BMP, PNG, and TIFF) required specific settings to be considered. For instance, since TIFF is a container format, multiple encoders could be used to encode images within it. We applied similar considerations to the other formats, focusing on settings that impact the entire file after conversion. For example, varying levels of compression alter the internal bytes of a file, making the resulting fragments distinct. All format-specific settings are detailed in the supporting document.

### Data augmentation

After examining the settings for each format, we observed an imbalance in our converted data. The JPEG format, in particular, had significantly more settings compared to the other four formats, requiring each file to be converted 78 times to ensure that each source image was processed with a diverse set of 10 different setting combinations. To balance the dataset, we applied horizontal flipping and rotations of 0, 90, 180, and 270 degrees to the remaining four formats for each content category.

### Conversion

Given the large number of source images and setting combinations, we utilized Python and the Pillow library to convert the images to the desired formats. Due to limitations in converting raw image formats, Nature images were first converted to full-quality JPEGs before being further processed into the desired format/settings using the Rawpy library. To cover a broad range of JPEG settings, each image was converted multiple times with random combinations of settings. Details of these settings are listed in the supporting document, which is available in Data file 16, Table 1.

### Taking fragments

Unlike many other file fragment datasets, such as those used by Fakouri and Teimouri [6], we retained all fragments from the beginning and end of the files. In cases where files did not contain enough fragments, we employed sampling with replacement. Additionally, when an end-of-file fragment was not long enough, the remaining portion was filled with random bytes to simulate a typical scenario where fragments are recovered from a hard drive. More information on the fragments can be found in the supporting document.

### Provided dataset

The dataset includes 15 data files in .dat format, with each file representing a unique format/content pair.

In addition to the data files, the supporting materials include results from machine learning models trained on the dataset (refer to the supporting document). The MATLAB source code for converting the fragment data files to CSV format, as well as the Python source code used in the conversion process, are also provided.

All of the mentioned files are available online as listed in Table 1.

**Table 1:** Overview of data file/files

Labels	Name of data file	File extension	Data repository
Data file 1	Nature_JPEG	Generic Binary Data (.dat)	Mendeley data [7] 10.17632/r9b362tthp.1
Data file 2	Nature_PNG	Generic Binary Data (.dat)	Mendeley data [7] 10.17632/r9b362tthp.1
Data file 3	Nature_GIF	Generic Binary Data (.dat)	Mendeley data [7] 10.17632/r9b362tthp.1
Data file 4	Nature_TIFF	Generic Binary Data (.dat)	Mendeley data [7] 10.17632/r9b362tthp.1
Data file 5	Nature_BMP	Generic Binary Data (.dat)	Mendeley data [7] 10.17632/r9b362tthp.1
Data file 6	Medical_JPEG	Generic Binary Data (.dat)	Mendeley data [7] 10.17632/r9b362tthp.1
Data file 7	Medical _PNG	Generic Binary Data (.dat)	Mendeley data [7] 10.17632/r9b362tthp.1
Data file 8	Medical _GIF	Generic Binary Data (.dat)	Mendeley data [7] 10.17632/r9b362tthp.1
Data file 9	Medical _TIFF	Generic Binary Data (.dat)	Mendeley data [7] 10.17632/r9b362tthp.1
Data file 10	Medical _BMP	Generic Binary Data (.dat)	Mendeley data [7] 10.17632/r9b362tthp.1
Data file 11	People_JPEG	Generic Binary Data (.dat)	Mendeley data [7] 10.17632/r9b362tthp.1
Data file 12	People _PNG	Generic Binary Data (.dat)	Mendeley data [7] 10.17632/r9b362tthp.1
Data file 13	People _GIF	Generic Binary Data (.dat)	Mendeley data [7] 10.17632/r9b362tthp.1
Data file 14	People _TIFF	Generic Binary Data (.dat)	Mendeley data [7] 10.17632/r9b362tthp.1
Data file 15	People _BMP	Generic Binary Data (.dat)	Mendeley data [7] 10.17632/r9b362tthp.1
Data file 16	Supporting_doc	Portable Document Format (.pdf)	Mendeley data [7] 10.17632/r9b362tthp.1
Data file 17	Conversion	Python Script (.py)	Mendeley data [7] 10.17632/r9b362tthp.1
Data file 18	Frag_extractor	MATLAB Script (.m)	Mendeley data [7] 10.17632/r9b362tthp.1

## Limitations

- **Formats**
  Our study focused exclusively on the five most common image formats (JPEG, BMP, GIF, PNG, and TIFF). While these formats are widely used, this selection may not represent the full spectrum of image file formats encountered in real-world scenarios. As a result, the findings may not be generalizable to less common formats that could exhibit different characteristics or behaviors in fragmentation and recovery.
- **Content**
  We chose only three categories of image content—Nature, People, and Health—based on their distinctiveness. This limited selection may overlook other important content types that could provide further insights into the performance of classifiers. Consequently, our results may not fully capture the diversity of image content encountered in practical applications of file fragment recovery.
- **Settings**
  The use of the Pillow library for image conversion introduced certain limitations, as not all possible effective settings may have been available for our dataset. This constraint might affect the diversity of fragments generated, potentially limiting the dataset’s robustness for training and evaluating machine learning models. Further research could explore additional settings or conversion libraries to enhance the dataset’s comprehensiveness.

## Data Availability

The data described in this Data note can be freely and openly accessed on Mendeley Data under the name ITC-MNP: A diverse dataset for image file fragment classification at 10.17632/r9b362tthp.1.

## Abbreviations

JPEG: Joint photographic experts group
BMP: Bitmap
GIF: Graphics interchange format
PNG: Portable network graphic
TIFF: Tagged image file format
